# Photocatalytic Activity and Mechanical Properties of Cements Modified with TiO_2_/N

**DOI:** 10.3390/ma12223756

**Published:** 2019-11-14

**Authors:** Magdalena Janus, Szymon Mądraszewski, Kamila Zając, Ewelina Kusiak-Nejman, Antoni W. Morawski, Dietmar Stephan

**Affiliations:** 1 Faculty of Civil Engineering and Architecture, West Pomeranian University of Technology, Szczecin, al. Piastów 50, 70-311 Szczecin, Poland; kamila.zajac@zut.edu.pl; 2 Building Materials and Construction Chemistry, Technische Universität Berlin, Gustav-Meyer-Allee 25, 13355 Berlin, Germany; szymon.madraszewski@tu-berlin.de (S.M.); stephan@tu-berlin.de (D.S.); 3 Faculty of Chemical Technology and Engineering, West Pomeranian University of Technology, Szczecin, ul. Pułaskiego 10, 70-310 Szczecin, Polandamor@zut.edu.pl (A.W.M.)

**Keywords:** photoactive cement, TiO_2_/N, NOx decomposition, mechanical properties

## Abstract

In this paper, studies of the mechanical properties and photocatalytic activity of new photoactive cement mortars are presented. The new building materials were obtained by the addition of 1, 3, and 5 wt % (based on the cement content) of nitrogen-modified titanium dioxide (TiO_2_/N) to the cement matrix. Photocatalytic active cement mortars were characterized by measuring the flexural and the compressive strength, the hydration heat, the zeta potential of the fresh state, and the initial and final setting time. Their photocatalytic activity was tested during NOx decomposition. The studies showed that TiO_2_/N gives the photoactivity of cement mortars during air purification with an additional positive effect on the mechanical properties of the hardened mortars. The addition of TiO_2_/N into the cement shortened the initial and final setting time, which was distinctly observed using 5 wt % of the photocatalyst in the cement matrix.

## 1. Introduction

In the last few decades, nanoparticles have been considered as an additive to the concrete and related cement products in order to improve the properties of building materials [1]. The first documented addition of the nanoparticles to a cement-based system occurred in 1964 when the nano-SiO_2_ facilitated a faster and more complete hydration of cement [2]. However, the application of various nanoparticles, such as nano-TiO_2_, nano-Al_2_O_3_, and nano-Fe_2_O_3_ in cement and concrete materials has developed intensively since circa 2004 [3,4,5]. Combining TiO_2_ nanoparticles with cementitious binders appeared to be one of the most promising ways to obtain environmentally friendly products [6]. Namely, a TiO_2_ photocatalyst, when activated by the suitable light, is capable of supporting the chemical reactions, which can degrade an atmospheric pollutant and give a self-cleaning property [7]. It is worth pointing out that the building surfaces are exposed to the highest levels of air pollution and at the same time to the solar radiation, which is necessary in photocatalytic processes. 

In the urban areas, NOx (NO + NO_2_) is one of the most common pollutants from the external sources (traffic, industry) [8]. NOx contributes to the formation of the photochemical smog, and it is associated with lung problems and asthma [9]. The potential of cementitious materials containing photocatalysts to decrease the NOx concentration was proven many times [10,11,12]. For example, Lee et al. [13] studied changes in NO and NO_2_ concentration using TiO_2_-containing cement-based materials during UV irradiation, suggesting that the materials are capable of oxidizing both gases efficiently. It was observed the similar amounts of NO and NO_2_ gases were degraded at 3 h, regardless of the variations in the water/cement ratio. The mechanism of NOx degradation consists of a series of reactions that take place during the photocatalytic process. Typically, it can be described as a sequential oxidation process, as follows: NO → HNO_2_ → NO_2_^−^ → NO_3_^−^ [8,11]. 

Many works in the photocatalytic branch are directed at modification of the base TiO_2_ structure through doping of the photocatalyst with the non-metals or the metal ions [14,15]. Mainly, it can enhance its activation in the visible light [16], but other advantages are also observed. In the treatment of NOx, modifications of TiO_2_ can improve the catalytic selectivity toward nitrate rather than the more toxic NO_2_ [7]. 

Although the photocatalytic cements and concretes have been extensively studied [17], it is still controversial whether the added photocatalyst enhances building properties. On the one hand, the presence of TiO_2_ nanoparticles can have a positive filler effect in cement mortars, increasing the mechanical strengths. One of the reported best performance enhancement results of the inclusion of TiO_2_ nanoparticles in the cementitious materials included a 45% increase in the compressive strength [18] and an 87% increase in the flexural strength [19]. Yang et al. [20] indicated that the addition of 0.5 wt % TiO_2_ to cement slag pastes allowed achieving approximately 10%, 15%, and 9% higher compressive strengths in comparison to the reference material at 3 d, 7 d, and 28 d, respectively. Meanwhile, the flexural strengths of the same photocatalytic materials were 25%, 25%, and 38% higher than the reference sample after the same range time of the curing. On the other hand, researchers also observed [21,22] a slight decrease in the mechanical strength of the photocatalytic cement mortars, which has been attributed to the decline of the sample’s homogeneity and the formation of weak zones in the structure of hardened mortars. 

Some authors [7] indicated that the effective use of photocatalysts in cement is highly connected with the assurance of the optimized dispersion of TiO_2_ particles in the cement matrix. The agglomeration of TiO_2_ particles can interfere not only with the mechanical strength, but also block access to an internal surface of TiO_2_, limiting the photocatalytic efficiency. The degree of repelling between particles of cement mortars has a direct relationship with “zeta potential”, which shows the electrokinetic behavior of particles and gives a valuable indication of the surface charge state, achieving values from −30 mV to +30 mV [23,24]. When the constituent particles of mortars have the same charge, they tend to repeal each other, and no agglomeration occurs. Up to now, the zeta potential measurements of cementitious materials have been performed with a low fraction of solid. Therefore, it is challenging to obtain information about the zeta potential values of real fresh cement mortars and even more so referring to photocatalytic cement mortars. Lowke and Gehlen [24] considered the zeta potential of Portland cement and mineral additions in cement suspensions with high solid fractions. They found a continuous increase in the zeta potential of cementitious suspensions with the increasing w/c ratio (water/cement). Moreover, the determining factor on the zeta potential appeared to be the molar Ca/SO_4_ concentration ratio, which was more crucial than the effect of the type of addition.

The absolute values of zeta potential may vary not only with mortar composition or w/c ratio but also with the time of hydration [25]. The hydration mechanism of cement consists of the reactions of cement components (e.g., alite or tricalcium silicate, belite or dicalcium silicate) with water. The formed crystalline calcium hydroxide and calcium–silicate–hydrate (C-S-H) comprise over 60 wt % of the hydration products in the total mass [26]. As the reactions continue with time, the hydration products gradually bind together and with other components of concrete to form a solid mass. The hydration of cement is an exothermic chemical reaction. The generation of heat is highly determined by the chemical composition of the cement mixture. It was reported that nanoparticles of TiO_2_ could significantly change the hydration of cement and influence the rates of heat evolution [27,28]. The cement hydration is directly related to the setting time of cement mortars. Mostly, the photocatalytic cements showed a shortened initial and final setting time for the samples with higher TiO_2_ contents, which is attributed to the acceleration of the hydration rate [29]. 

The aim of this paper is to present the results of our study on the influence of a prepared TiO_2_/N photocatalyst on the properties of the fresh and hardened cement mortars. TiO_2_/N was chosen as the photocatalyst because there is the possibility of producing this material in the amount of 0.5 kg per day. Moreover, the technological project of installation for TiO_2_/N production exists and may be used for photocatalyst production at a large scale. The photocatalyst has been added in different dosages (1, 3, and 5 wt % to cement mass) to cement mortars. The measurements of the initial and the final setting time, the flexural and the compressive strength, the hydration heat, and the zeta potential were conducted. The photocatalytic activity was monitored during the NOx degradation process.

## 2. Materials and Methods 

### 2.1. Materials

Ordinary Portland cement CEMI 42.5 N from Holcim, Germany was used in this study. Standard sand, according to EN 196-1, was used for all mortars. 

The preparation of the photocatalyst (TiO_2_/N) was carried out using HEL Ltd. ‘‘Autolab’’ E746 installation. The commercial titanium dioxide supplied by Grupa Azoty Zakłady Chemiczne ‘Police’ S.A. (Poland) was used as a starting material. First, 600 g of TiO_2_ and 350 mL of NH_4_OH with a concentration of 2.5% were placed in an autoclave. The reactor was closed, and the mixture was blended using a magnetic stirrer and heated up to 100 °C for 4 h. Afterwards, the catalyst was dried in air for 4 h at 100 °C. Finally, the obtained photocatalyst TiO_2_/N was ground with mortar to form a fine powder. The structural and the textural parameters on N-modified TiO_2_ in Table 1 were placed. The results of TEM (transmission electron microscope), XRD (X-ray powder diffraction), FTIR/DRS (fourier transform infrared spectroscopy/diffuse reflectance), XPS (X-ray photoelectron spectroscopy), and Raman spectroscopy in our earlier publication were presented [30]. The presence of nitrogen in the modified titania sample was confirmed by FTIR analysis. The narrow bands at 1640 cm^−1^ and 1440 cm^−1^ are attributed to the hydroxyl (OH) and ammonium (NH_4_^+^) groups, respectively, while the band at 1536 cm^−1^ could be assigned to either NH_2_ or NO_2_ and NO groups. The sample was also studied by Raman spectroscopy. The Raman spectra of the sample exhibit four distinct peaks located at 145 cm^−1^, 393 cm^−1^, 514 cm^−1^, and 646 cm ^−1^; those bands correspond to the anatase phase of TiO_2_ [30].

### 2.2. Specimen Preparation

The specimens 40·40·160 mm^3^ and 80·40·10 mm^3^ were produced according to EN 196-1 with a water to binder ratio (w/b) = 0.4 and cement to standard sand ratio of 1:3. Cement was replaced by catalyst in 1, 3, and 5 wt % by mass of cement. Samples without replacement were produced as a reference. For each type of mortar, 6 specimens were produced. Masses needed for the preparation of 3 specimens are presented in Table 2.

A standard mixer with a stainless steel bowl with a capacity of 5 dm^3^ according to EN 196-1 was used. First, water was poured into a bowl, and cement was added. The mixer was started immediately at low speed (rotation 140 min^−1^) and after 30 s, standard sand was steadily added for the next 30 s. Afterwards, the mixer was switched to the higher speed (285 min^−1^) for an additional 30 s. To remove all the mortar adhering to the wall and the bottom part of the bowl, the mixer was stopped for 90 s. In the end, the mixing was continued at high speed for 60 s. The specimens were molded immediately after the preparation. The first layer of mortar was poured into the mold situated on the jolting table and then compacted. The second layer of mortar was poured on the first layer and compacted. The excess mortar was struck off with the straight metal edge. Casting molds containing fresh samples were wrapped with stretch film and stored at room conditions for 24 h. All specimens were demolded after 1 day and were cured in tap water for the next 27 days.

### 2.3. Compressive and Flexural Strength Measurements

After 28 d, specimens were tested for their flexural and the compressive strength. The flexural and the compressive strength measurements were carried out following EN 196-1. For each mortar type, six 40·40·160 mm^3^ specimens were tested for the flexural strength. The prism halves (after the test of flexural strength) were tested for compressive strength, so for each mortar type, 12 specimens were tested. A standard testing machine (ToniNORM 2010.040, Toni/Technik, Berlin, Germany) was used both for the flexural and the compressive strength measurements.

### 2.4. Setting Time (Vicat Needle Test)

The setting of cement and its rate affects the open time of the mortar. In this study, the influence of the addition of the catalyst on the setting time of cement was tested. The Vicat Apparatus is a device that is used to determine the setting time of the cement paste. In this study, an automatic device ToniSET COMPACT version 05/00, which did 6 parallel tests, was used for determining the setting time. For each mortar type, 2 specimens were tested. Mortar preparation and the setting time measurements were run according to the EN 196-3 standard. During the measurement, the specimens were kept at 20 °C. The water to binder ratio of paste used for the setting time test was w/b = 0.3. The time when the needle stopped 6 mm from the base plate was recorded as the time for the initial setting. The final setting was defined as the time when the needle only made a 0.5 mm mark on the surface. 

### 2.5. Hydration Heat Measurements

Calorimetry data were obtained from externally mixed pastes containing 40 g of cement and 16 g of water, in at least a twofold determination. Data points were recorded every 60 s at 20 °C (Isothermal heat flow calorimeter MC-CAL100, *C3 Analysentechnik*, C3 Prozess und Analystechnik, Haar, Germany).

### 2.6. Zeta Potential Measurements

A Zeta and Titration 310 instrument from Dispersion Technology was used for the zeta potential measurements without the dilution of samples, which to some extent avoided the differences in the hydration and surface properties between diluted and original samples. First, 16 g of water was added to 40 g of cement and mixing for 20 s. As the background, the centrifuged water from such prepared mortars was used. The average particle sizes of cement amounted to 9 µm. 

### 2.7. NOx Decomposition

**Figure 1:**
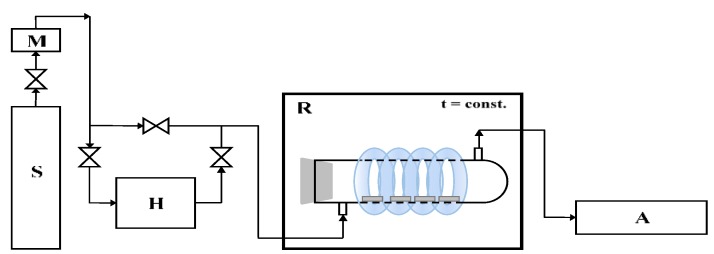
The scheme of installation to the photocatalytic removal of NOx (S—the source of pollution; M—mass flower; H—humidifier; R—photocatalytic reactor with irradiation source; A—NOx analyzer).

The photocatalytic activity of the prepared plates of cement mortar toward the degradation of air pollutions was also proved. In our previous works [31,32], the gaseous NO (1.989 vppm ± 0.040 ppm, Air Liquide) was used as model pollution in photocatalytic tests. NOx removal was evaluated using the experimental installation, whose scheme is presented in Figure 1.

The studied plate of cement mortar (one at dimensions of 80 × 40 × 10 mm^3^) was placed in the central part of a cylindrical reactor (Pyrex glass; Ø × H = 9 × 32 cm^2^), and the reactor was tightly closed. The NO was diluted with humidified synthetic air in a ratio of 1:1. The oxygen and water molecules were necessary for the formation of oxidative species, which are essential in the photocatalytic reactions. The polluted air flowed through the reactor continuously with a rate of 500 cm^3^/min. At the beginning of the process, the dark conditions were maintained until NO concentration reached equilibrium (about 1 ppm during about 35 min). Then, the UV lamps were turned on for 30 min. The irradiation sources surrounded the reactor and were characterized by the cumulative intensity of 100 W/m^2^ UV and 4 W/m^2^ VIS. The temperature of the whole system was stable at the level of 22 °C by using a thermostatic chamber. The NO and NO_2_ concentrations were continuously measured in the outlet of the reactor using chemiluminescent NOx analyzer (T200, Teledyne). All measurements were repeated three times, and errors were 2%.

## 3. Results

### 3.1. Compressive and Flexural Strength

**Figure 2:**
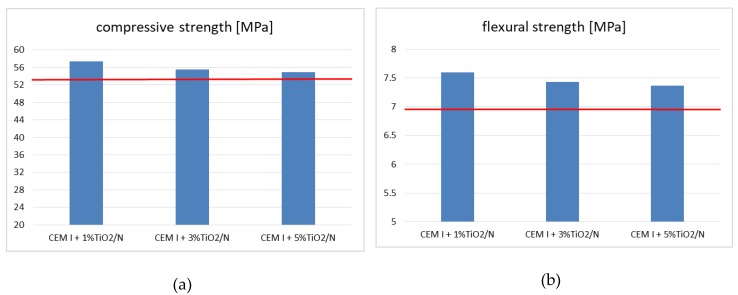
(**a**) Compressive and (**b**) flexural strength of CEM I 42.5 N with the addition of 1, 3, and 5 wt % of photocatalyst TiO_2_/N. In the red line, the compressive strength (53 MPa) and flexural strength (6.92 MPa) of pure CEM I 42.5 was presented.

The compressive and the flexural strength of pure cement and cement with the addition of 1, 3 and 5 wt % TiO_2_/N specimens were measured. The obtained results are presented in Figure 2a,b. As it can be seen in Figure 2a, the value of the compressive strength of unmodified cement amounted to 53 MPa (red line), while the addition of 1, 3, and 5 wt % of TiO_2_/N increased the compressive strength of the specimens in all cases. The highest value of the compressive strength was observed for specimens with 1 wt % of TiO_2_/N and amounted to 57.4 MPa. The lowest increase of the compressive strength was found for a specimen with the addition of 5 wt % of TiO_2_/N. Similar behaviour occurred during the flexural strength measurements. As can be seen in Figure 2b, the value of the flexural strength of unmodified cement amounted to 6.92 MPa (red line). Analogous as in the case of the compressive strength, the addition of 1, 3 and 5 wt % of TiO_2_/N increased the flexural strength of the specimens in all cases. The highest value of the flexural strength was observed for a specimen with 1 wt % of TiO_2_/N and amounted to 7.60 MPa. The lowest increase of flexural strength was obtained using specimen with addition of 5 wt % of TiO_2_/N.

The mechanical properties (the compressive and the flexural strength) of cement strongly depend on the amount of used titanium dioxide. Wang et al. [33] discovered that with the incorporation of TiO_2_ nanoparticles, the strength firstly showed a fast increase compared with the ordinary mortar until the dosage of TiO_2_ nanoparticles reached up to 2 wt %, and then the rate of this increase slowed down. The strength of the cement mortar is closely related to the amount of ettringite and C-S-H gels, and the existence of nanoparticles facilitates the cement hydration, thereby producing more hydration products. In addition to the filler property of nanoparticles to fill the pores in C-S-H gels, it is well known that nanoparticles have a large surface area to volume ratio, and hence, the additional surface area turns out to be an appropriate place for hydration products to precipitate. Nanoparticles enable the formation of a bond between itself and C-S-H gels. As a result, the strength can be accordingly improved. However, there is also an undesirable effect due to the large ratio of surface area to volume, since nanoparticles can glue together, many nanoparticle clusters, and the strength that can be generated is very weak, leading to a heterogeneous microstructure. 

Beyond 3 wt % nano-TiO_2_, the cementing system seems to be saturated, and the poor dispersion of the nanoparticles generated by their high surface area may create weak zones in the system. In addition, it could also enhance the particle packing density of the blended cement by filling up the nanopores and reducing both the larger pores as well as the overall porosity of the mix. This decreased the total specific volume of the pores; the refinement of the pore structure when up to 3 wt % nano-TiO_2_ is used as a partial replacement of cement was also reported by Praveenkumar et al. [34] and Nazari and Riahi [35,36]. 

### 3.2. Setting Time

The initial and the final setting time of tested specimens is presented in Table 3. As can be seen, with the increasing addition of TiO_2_/N to cement, the initial setting time decreased. In the case of the specimens modified by the addition of 5 wt % of TiO_2_/N, the initial setting time was 40 min faster than that for unmodified cement. The same behavior was observed for the final setting time. With an increasing addition of TiO_2_/N to cement, the final setting time decreased. Specimens of cement with an addition of 5 wt % of TiO_2_/N showed a final setting time that was about 57 min faster in comparison to the unmodified cement. A similar observation was made by Hernández-Rodríguez et al. [37]; they added commercial TiO_2_ P25 to CEM I 52.5 R and the results showed that the photocatalysts act as a setting accelerator.

### 3.3. Hydration Heat

**Figure 3:**
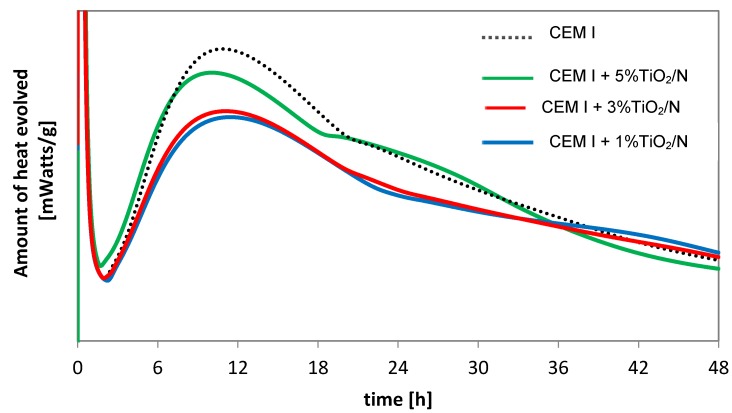
Isothermal calorimetry results for cement modified by the addition of 1, 3, and 5 wt % of TiO_2_/N to deionized water at a water to binder ratio (w/b) = 0.4.

In Figure 3, the isothermal calorimetry results of unmodified cement and cement modified by the addition of 1, 3, and 5 wt % of TiO_2_/N photocatalysts were presented. 

According to the literature, there are five stages of heat for a typical Portland cement [38,39]. The addition of modified titanium dioxide into the cement influences hydration heat; the paste with the addition of TiO_2_/N showed less heat generated up to 20 h compared to the unmodified cement.

### 3.4. Zeta Potential Measurements

The average value of zeta potential amounted to −5.01 mV for unmodified cement mortar and −4.90 mV, −4.69 mV and −5.94 mV for cement mortars modified by the addition of 1, 3, and 5 wt % of TiO_2_/N, respectively.

It is worth pointing out that TiO_2_ photocatalysts are characterized by a negative charge in high pH medium. In our previous work [40], it was proven that the point of zero charge of TiO_2_/N is about 5.8. Namely, the TiO_2_/N surface appeared to be positively charged at pH < 5.8, whereas it was negatively charged at pH > 5.8. The application of TiO_2_/N with highly alkaline cement resulted in the presence of a negatively charged form of TiO_2_/N particles.

Zingg et al. [41] concluded that the phases C_3_S and C-S-H are positively charged, whereas the ettringite is negatively charged. During the initial stage of cement hydration, the aluminate reacts with water and sulfate, forming a gel-like material (ettringite) surrounding the cement grains. The negative values of zeta potential at the beginning of the hydration process confirmed it. 

### 3.5. NOx Decomposition

**Figure 4:**
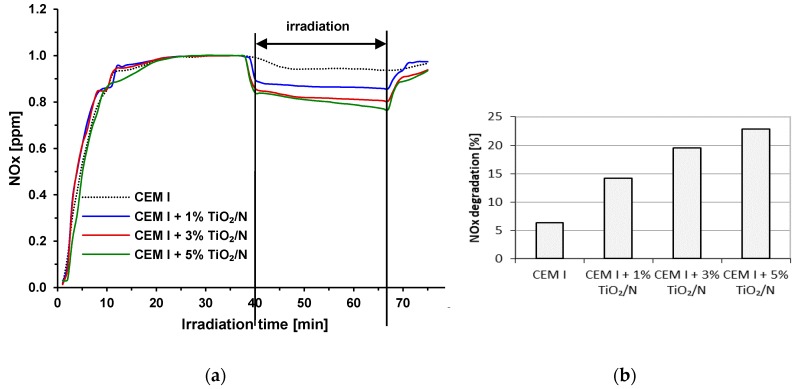
(**a**) Graph of NOx [ppm] decomposition and (**b**) NOx degradation [%] on CEM I samples, and cements modified by the addition of 1, 3, and 5 wt % of TiO_2_/N under UV light irradiation.

In Figure 4, the photocatalytic activity of unmodified and modified cements is presented. The activity of obtained materials during NO removal was tested. The mechanism of photocatalytic NO removal is as follows [42]. Initially, active oxidizing groups are generated at the TiO_2_ surface (reactions 1–3):

O_2_ + e^−^ → O_2_^−^ (1)

OH^−^ + h^+^ → ·OH (2)

H^+^ + O_2_^−^ → HO_2_. (3)

The action of these moieties on NO molecules leads to their oxidation to the form of NO_2_, followed by the formation of nitric(III) and (V) acids (reactions 4–6):

NO + HO_2_^−^ → NO_2_ + ·OH (4)

NO_2_ + ·OH → HNO_3_ (5)

NO + ·OH → HNO_2_. (6)

In Figure 4a, the decreasing of NOx concentration [ppm] is presented. During the first 40 min, the equilibrium of NO was obtained. After 40 min, the UV light was switch on, and it is possible to observe that the NOx concertation decreased. The irradiation takes 30 min, and after this time, the light was switched off. Figure 4b presents the NOx degradation in percent after 30 min of UV light irradiation. The reference sample, pure CEM I, showed the removal of NOx on the level of about 6.3%. The same observation concerning the blank sample was presented by Xu et al. [43]. They found that using reference cement composites without any TiO_2_, the NOx concentration slowly decreased by 6% during 15 min of irradiation. It is worth pointing out that in our studies, the photolysis of tested gas amounted to 1.3% under the same conditions and the same irradiation source. The application of nitrogen-modified TiO_2_ in cement mortars involved the degradation of NOx on the photocatalytic path, which can be observed as the unambiguous decrease of NOx concentration directly after turning on the irradiation. The increase of TiO_2_/N loading in cement matrix caused the increase of the NOx degradation rate from 14.2% for CEM I + 1 wt %TiO_2_/N to 22.9% for CEM I + 5 wt %TiO_2_/N. Apart from the influence of the photocatalyst dose in the cement matrix, the accessible surface area of the photocatalyst is essential for the photocatalytic effectiveness [10]. Therefore, we did not observe a proportional increase of NOx degradation rate with the higher TiO_2_/N loading. However, it appeared that the nitrogen-modified photocatalyst might be used as an additive to cement materials to increase its air purification properties. Moreover, in Table 4, the NO removal and NO_2_ formation during the photocatalytic oxidation of NO are presented. 

In Table 5, the initial photodegradation rates are presented. It was calculated 5 min after switching on the UV light. This value was calculated as µg of NO removal, NO_2_ creation, and NOx total removal on the surface of modified cement plates [cm^2^] during the time of UV light irradiation [h]. As it can be seen, the highest vales of NO removal, NO_2_ creation, and NOx total removal were when the cement was modified by the addition of 5 wt % of TiO_2_/N.

The similar results of NOx photocatalytic degradation on cement materials were observed by other authors as well. It was reported [13] that 5% TiO_2_ replacement by the mass of cement in cement pastes allowed decreasing the NO concentration from 1 ppm to about 0.7 ppm. The results were calculated after 3 h of exposure to UV irradiation, because it was the necessary time to achieve the relative stasis in NO concentration. Jimenez-Relinque et al. [21] applied 2% of commercial TiO_2_ with different types of cement in normalized mortars. NO gas diluted in the air was used as model pollutant with an initial concentration of 1 ppm ± 50 ppb. After 1 h of UV irradiation, they obtained NO photocatalytic degradation on the level of 15–30% and NOx removal in the range of 18–25%, depending on the applied cement type.

**Figure 5:**
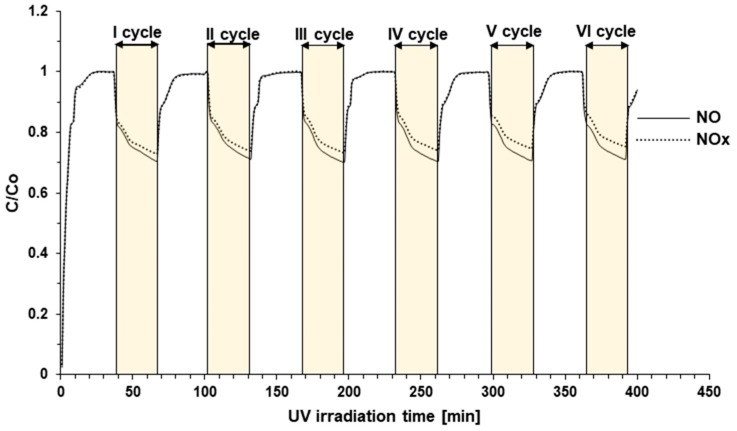
The lifetime of CEM I + 5 wt %TiO_2_/N under six cycles of irradiation.

In Figure 5, the lifetime of tested modified cement plates was presented. As it can be seen, there was no decrease in the photocatalytic activity of the modified cement plates. NO removal and total NOx removal are on the same level. There are only small differences between NO and NOx concentration, and this behavior suggests that with time (increasing the number of cycles), more NO_2_ is produced. 

## 4. Conclusions

The nitrogen-modified titanium dioxide (TiO_2_/N) may be used as an additive to cement mortars to produce the cement with photocatalytic properties. All photocatalytic samples degraded regarding the NOx concentration during irradiation time, achieving a higher NOx removal rate with a higher TiO_2_/N dosage in cement materials. The addition of TiO_2_/N up to 5 wt % into the cement mortar did not decrease the mechanical properties but even slightly increased the compressive and the flexural strength. 

Nanoparticles of TiO_2_/N appeared to have an influence on the cement hydration. Acceleration of the initial and the final setting time indicated that the photocatalytic particles might act as seeds for the precipitation of C-S-H. The addition of 5 wt % of TiO_2_/N into the cement mortar shortened the setting time by about 57 min. Moreover, the presence of TiO_2_/N in the cement matrix caused less heat to be generated during the hydration process. 

The negative charge of high solid cement mortar, which was determined based on the zeta potential, was amplified using a higher amount of TiO_2_/N photocatalyst, from –4.3 mV to –5.5 mV at the beginning of hydration. High TiO_2_/N loading in the cement matrix resulted in more negative zeta potential, because the very fine TiO_2_ is negatively charged at a high pH.

## Figures and Tables

**Table 1:** Structural and textural parameters of N-modified TiO_2_.

Photocatalyst	Local Mean Crystallite Size According to TEM [nm]	Global Mean Crystallite Size According to XRD [nm]	Mean Particle Size According to DLS (Dynamic Light Scattering) [nm]	S_BET_ [m^2^/g]
TiO_2_/N	6.1	10.8	167.6	235

**Table 2:** Mass of materials used for the production of three 40·40·160 mm^3^ mortar specimens.

Materials	Mass of Used Materials [g]		
	1%	3%	5%
CEM I 42.5 N	444.5	436.5	427.5
TiO_2_/N	4.5	13.5	22.5
Standard sand	1350	1350	1350
Water	180	180	180

**Table 3:** The values of initial and final setting time of CEM I and CEM I with the addition of 1, 3, and 5 wt % of TiO_2_/N photocatalysts.

Samples	The Initial Setting Time [min]	The Final Setting Time [min]
CEM I 42.5N	218	305
CEM + **1** wt %TiO_2_/N	217	310
CEM + **3** wt %TiO_2_/N	207	275
CEM + **5** wt %TiO_2_/N	178	248

**Table 4:** The NO removal and NO_2_ creation during NO photooxidation with cement modified by TiO_2_/N.

Sample	NO Removal [ppm]	NO_2_ Formation [ppm]	NOx Removal [ppm]
Photolysis	0.023	0.013	0.010
CEM I	0.057	0.009	0.048
CEM I + 1% TiO_2_/N	0.141	0.030	0.111
CEM I + 3% TiO_2_/N	0.179	0.025	0.154
CEM I + 5% TiO_2_/N	0.211	0.032	0.179

**Table 5:** The initial photodegradation rate for modified cement during NO removal, NO creation, and NOx total removal.

Sample	NO Removal [µg/cm^2^/h]	NO_2_ Formation [µg/cm^2^/h]	NOx Total Removal [µg/cm^2^/h]
Photolysis	0.289	0.038	0.251
CEM I	0.315	0.091	0.224
CEM I + 1% TiO_2_/N	2.530	0.622	1.908
CEM I + 3% TiO_2_/N	3.145	0.496	2.649
CEM I + 5% TiO_2_/N	3.403	0.651	2.752
